# Supplementary material for: Are carbon emissions trading and green financial instruments synergistic? -Comprehensive quantitative research based on content analysis
Source: PLoS One. 2024 Mar 7;19(3):e0298601. doi: 10.1371/journal.pone.0298601 (PMC10919601; doi:10.1371/journal.pone.0298601)
Supplement: S1 Data — (ZIP) [file pone.0298601.s001.zip › Data/地级市Z_F工作报告.zip!/║■▒▒/╛ú╓▌╩╨/└┤╫╘╣1⁄2╓┌║┼í╛┬φ┐╦╩2╛▌═°í┐_2014.docx]

各位代表：

现在，我代表市人民政府向大会作工作报告，请予审议，并请各位政协委员和列席人员提出意见。

一、2013年工作回顾

过去的一年，面对错综复杂的经济形势和高温干旱等自然灾害，在省委、省政府和市委的坚强领导下，在市人大、市政协的监督支持下，我们紧紧依靠全市人民，克服重重困难，积极务实作为，荆州发展呈现新气象、大气势、好气场：地区生产总值达到1334.9亿元、增长10.4%，总量继续保持全省第四，增幅高于全省平均水平；固定资产投资、规模以上工业增加值、地方公共财政预算收入实现“三年翻番”，农产品加工成为首个千亿级产业；招商引资、项目建设、征迁安置、信访维稳等一线战场全面铺开，城市新区、工业园区、交通水利和政务服务体系建设取得重大突破。可以说，壮腰工程正在全面发力，已经从战略化布局进入战术化安排、战场化实施的关键阶段。

（一）着力推进产业扩张，经济发展呈现稳中向好之势。

工业经济逆势而进。克服经济增长下行压力，全市规模以上工业实现增加值530.5亿元、增长13.9%，增幅领跑全省市州。规模以上企业突破1000家、净增153家；销售收入10亿元以上企业22家，福娃集团、中兴能源、楚源集团分别达到82亿元、75亿元和70亿元，拍马集团突破60亿元；纳税亿元以上工业企业5家，白云边酒业税收达到6.66亿元。石油钻采设备产业纳入全国老工业基地调整改造规划，拍马林浆纸印刷包装纳入省重点扶持产业集群。农产品加工业产值1019亿元，装备制造、高新技术、医药化工产业分别突破400亿元、300亿元、200亿元，培育大产业迈出坚实的一步。

农业基础不断巩固。全力抗旱夺丰收，粮食实现“十连增”，总产77.2亿斤、增3亿斤。油菜总产1220万担，淡水产品产量122万吨，均稳居全国市州第一。新增省级以上农业产业化龙头企业22家，新型农业经营主体突破4000家，土地流转面积达到151万亩。监利县获批全国现代农业示范区改革与建设试点县，石首市被授予“全国蔬菜标准化建设示范县”。新增洪湖浪、小胡鸭等5个中国驰名商标，新增国家农产品地理标志和地理标志证明商标19个，“荆州鱼糕”荣获“国家地理标志产品”称号。争取水利建设、土地整理、农业开发项目资金31.6亿元，其中水利建设投资21.3亿元、为建国以来投入最多的一年。

服务业稳健发展。实现社会消费品零售总额738.3亿元、增长13.5%，居民消费价格涨幅3.1%。南国城市广场正式营业，两湖绿谷农产品交易额突破300亿元。烟草市场经营秩序进一步规范。外贸出口11.3亿美元、增长22.2%。熊家冢考古遗址公园通过国家评审验收，周老嘴红色革命旧址群建成开放，九老仙都、松滋洈水、洪湖岸边是家乡等景区建设加快。全市旅游综合收入111.9亿元、增长21.6%。汉口银行入驻荆州，6家县级农信社改制重组为农商行，全市新增银行贷款159.3亿元，连续六年被评为省“A级信用市州”。

（二）着力推进投资增量，开放开发呈现日趋强劲之势。

招商引资质效提升。实际到位资金740亿元，增长51%。新批外商投资企业11家，实际利用外资1.08亿美元、增长20%。引进亿元以上工业项目180个，开工率达40%。华讯方舟、华中塑胶、珠江实业精密铸锻、长江液晶显示屏、中联重科专用车、恒信汽车产业园、江陵斯米克新型建材、监利永康五金产业园、松滋华群新材料、石首中陶地板、洪湖雪花秀化妆品等一批投资10亿元以上的重大项目相继入驻，荆州振兴的后劲不断增强。

园区建设步伐加快。荆州开发区深圳大道、荆沙大道延伸线建成通车，“三纵七横”路网格局初步形成；华中农高区获批“国家农业科技园”，农高大道、太湖大道路基工程基本完工；江陵县沿江产业园、松滋市临港工业园、公安县青吉工业园、石首市金平工业园、监利县城东工业园、洪湖市新滩工业园等建设明显提速。全市开发区基础建设投入突破50亿元，建成区面积超过180平方公里，入园企业达到2551家、新增371家。油气钻采设备质量监督检验中心获批筹建，将成为荆州首个国家公共检验检测平台。

项目建设卓有成效。完成固定资产投资1355.6亿元、增长30%，其中工业投资665.9亿元、占投资总量的49.1%。伟特电子、同洲电子、群力金属、福娃六厂、中哈能源、白云边生态科技产业园、吉象CPS生产线、劲牌酒业技改扩能等项目年度投资均超过5亿元，法雷奥汽车空调热交换器、新动力电机、沙市钢管二期、奥达新厂、凯乐新型光纤、宜化肥业、家和宝厨具等一批项目竣工投产，即将成为荆州经济新的增长点。

（三）着力推进改革创新，发展环境呈现不断优化之势。

各项改革稳步实施。文化广电、文物旅游、卫生计生、食品药品和运管物流“大部制”改革全面完成，环保、人社等部门“大科室制”改革取得进展，事业单位分类改革正式启动，“大城管”格局初步形成。国资监管水平进一步提升，出资企业利润总额2.7亿元、增长14.3%。“营改增”试点改革稳步推进，财政整合资金、竞争分配、绩效管理机制逐步建立。监利新沟镇、洪湖峰口镇行政体制改革试点扎实开展，福娃模式成为全省“四化同步”发展的一面旗帜。

自主创新能力增强。新增校企共建研究中心2家、省级工程技术研究中心3家、院士专家工作站7家，长江大学新农村发展研究院挂牌成立。高新技术企业达到75家、新增15家。争取中省重大科技专项9项，申请专利1499个。四机厂企业技术中心获国家认定，凯乐集团成为全国光电缆行业标准制定企业。实施“招硕引博”工程，引进高层次管理、技术人才81人。我市被授予“全国科技进步考核先进市”称号，荆州区、松滋市、公安县获评“全国科技进步考核先进县”。

政务服务全面提速。行政审批“三集中、三到位”基本完成，企业登记注册“一表通”、收费项目“一表制”、工业项目审批服务“一条龙”全面实施，政务服务体系重构、行政审批流程再造取得实质性成效。清理减少行政权力事项1413项，下放行政审批权138项，工业项目审批环节由67个减至23个。争取用地指标2.68万亩，盘活闲置低效用地6640亩，重大项目供地得到有力保障。治庸问责深入开展，广大干部谋发展、促发展意识明显增强，加快振兴的合力已经形成。

（四）着力推进基础建设，城乡面貌呈现加快改善之势。

城市建设力度空前。沙北新区、荆北新区、华中农高区建设掀起新高潮，大城市框架加快形成。中心城区市政投入突破30亿元、是上年的两倍，新建改造城市道路78.3公里，新增城市绿地面积53.1公顷。明月公园、滨江公园初步建成，万达广场、绿地之窗、卓尔国际城等城市综合体项目相继开工。省运会筹备工作扎实推进，体育中心、奥体中心等主体工程基本完工。沙市区“阳光征迁、和谐征迁”刷新“荆州速度”，全市征迁面积超过320万平方米。城市创建活动深入开展，江陵县、监利县荣获“省级园林县城”称号。

农村条件逐步改观。荆州区、沙市区城郊乡镇控制性详细规划编制完成，松滋刘家场、石首桃花山、洪湖瞿家湾等中心镇和特色镇完成基础设施投资1.34亿元。洪湖滨湖成功创建“国家级生态乡镇”，公安斗湖堤、松滋新江口等5个乡镇入选“全省百强乡镇”。“无线城市”建设向农村延伸，城乡电网改造完成投资14.6亿元。通村公路新建1186公里，农村安全饮水人口新增32万人，农村危房改造7916户，村级垃圾清运体系实现全覆盖，美丽乡村建设步伐加快。

交通建设全面铺开。江南高速、洪监高速、潜石高速、公安东岳庙至卷桥高速、武汉城市圈环线荆州段等5条高速公路同步施工，荆松一级公路建设顺利推进；荆州长江公铁两用桥开工建设，石首长江大桥可研报告通过国家发改委咨询评估；引江济汉通航工程、荆州港涉外旅游码头、松滋车阳河综合码头等5个港航项目基本建成，港航建设迈入全省第一方阵。全市交通建设完成投资80.9亿元，大交通格局初具雏形。

（五）着力推进公共服务，社会事业呈现协调发展之势。

社会保障逐步完善。全市实现财政收入113亿元，地方公共财政预算收入71.95亿元、增长26.8%；地方公共财政预算支出255.9亿元，其中民生支出197.7亿元、占比达到77%。城镇居民人均可支配收入、农民人均纯收入分别达到18706元和9909元，增长10%和13.8%。新增城镇就业8.47万人，新增农村劳动力转移就业5.23万人。居民养老保险实现全覆盖，企业退休人员基本养老金标准由人均每月1296元提高到1446元；城乡居民大病医保全面启动并兑现赔付，住院最高支付限额由10万元提高到15万元。房地产市场逐步规范，保障性住房竣工18740套，发放租赁补贴1907户。社会救助体系进一步健全，城市低保标准由每月340元提高到390元，农村低保由每年1680元提高到1900元。

社会事业全面进步。新建学校9所、改扩建学校131所，松滋市通过县域义务教育均衡发展国家督导评估，荆州区、洪湖市通过义务教育均衡发展省级验收。“小太阳读书节暨全民阅读活动”成为国家公共文化服务体系示范项目。全民健身活动蓬勃开展。“幸福计生”创建不断深入，出生人口政策符合率93.58%。血吸虫病防治顺利通过国家传播控制标准考核。洪湖湿地获中央电视台“美丽中国·魅力湿地”特别关注奖，天鹅洲获批国家重要湿地，洈水成为国家湿地公园。植树造林23.9万亩，松滋市、公安县被评为“全国绿化模范县”。全面完成省下达节能减排任务，单位生产总值能耗下降3.9%。妇女儿童、老年人和残疾人合法权益得到保护，我市获批全国未成年人社会保护试点城市。国防动员、国防教育、人民防空和民兵预备役工作得到加强。扶贫开发、外事侨务、民族宗教、台湾事务、邮政通讯、防震减灾等工作取得新成绩，统计、供销、盐务、检疫、移民、农垦、气象、社科、史志、档案、保密和无线电等工作取得新进步。

社会管理不断加强。中心城区网格化管理有效运转。文明创建活动深入开展，大爱夫妻黄宏林、严玉芹荣获第四届全国道德模范提名奖，洪湖湖区医生谢爱娥夫妇当选“中国十大最美乡村医生”。公共安全体系不断完善，公众安全感、治安满意度和执法满意度得到提升，安全事故起数和受伤人数分别下降1.28%、16.4%。信访联合接访和矛盾纠纷大调解机制逐步完善，群众诉求渠道更加畅通。依法行政扎实推进，政务公开、政务督查、新闻发布、应急管理得到加强。向人大报告工作、向政协通报情况制度化、常态化，自觉接受人大法律监督、工作监督和政协民主监督，虚心听取民主党派、工商联和无党派人士意见，办理人大代表建议105件、政协提案298件。十件实事全面完成。贯彻中央八项规定、省委六条意见和市委七条意见收到实效，审计监督和行政监察不断强化，一批违纪案件得到查处。“转作风、抓落实、晒实绩”主题活动扎实开展，真抓实干、争先进位的正能量不断汇聚。

各位代表！今天的荆州，发展态势令人鼓舞，发展气势催人奋进，发展前景让人憧憬！荆州发展的每一步、每个变化，都得益于省委、省政府和市委的正确领导，得益于市人大、市政协和社会各界的监督支持，得益于全市人民的共同努力。在此，我代表市人民政府，向所有关心和支持荆州发展的同志们、朋友们，致以诚挚的感谢和崇高的敬意！

各位代表！当前的荆州，正处于历史上最好的发展时期，也是发展责任与社会矛盾交织、工作难度空前的时期，我们务必时刻保持清醒、理性，深刻认识到发展中仍存在一些不容忽视的问题：受宏观环境影响，生产总值、规模以上工业增加值、社会消费品零售总额三项指标增幅未能达到年初预期目标；产业结构不够合理，农业基础仍很脆弱，工业竞争力不强，服务业增长缓慢，县域经济块头较小；地方可用财力有限，城乡建设欠账较多，城市管理、环境保护、民生事业等方面仍不尽人意，部分群体生活还比较困难；少数部门创新意识、担当意识、服务意识有待进一步提升。问题并不可怕，落后不会永恒。只要我们站在历史的高度，把握时代的脉搏，勇担振兴的责任，辩证看、务实办、齐心干，荆州就一定能创造属于自己的未来！

二、2014年工作任务

2014年，是全面深化改革的开局之年，是壮腰工程“三年见成效”的攻坚之年。做好今年的工作，事关改革大局，事关振兴全局。

今年政府工作的总体要求是:认真贯彻落实党的十八大、十八届三中全会、中省经济工作会和市第四次党代会精神，坚持“稳中求进、改革创新”总基调和“竞进提质、升级增效”总要求，把改革创新贯穿经济社会发展各个领域各个环节，扩大投资增量，推动转型升级，优化发展环境，激发市场活力，增进民生福祉，努力完成壮腰工程“三年见成效”阶段性任务，为振兴荆州打下坚实基础。

今年经济社会发展的预期目标是：生产总值增长10.5%，固定资产投资增长26%，规模以上工业增加值增长14%，社会消费品零售总额增长14%，外贸出口增长12%，地方公共财政预算收入增长15%，城镇居民人均可支配收入增长10.5%，农民人均纯收入增长10.5%，居民消费价格涨幅控制在4%左右，城镇登记失业率控制在4.5%以内，人口自然增长率控制在6.5‰以内，完成好省下达的节能减排任务。

在目标的制定上，我们是审慎的，也是积极的。既积极响应中央关于全面深化改革、加快转型升级的总体部署，也积极回应社会各界的期盼和广大市民的关切；既充分考虑宏观形势可能造成的不利影响，也充分体现壮腰工程“三年见成效”的现实要求。应该看到，荆州经济持续向好的态势已经形成，后发赶超的条件已经成熟，正处在应有作为、大有作为的战略机遇期。

我们务必把握大势，改革精进。中央全面深化改革的力度前所未有，决心前所未有。当前，发展的外部环境更加复杂，一系列体制机制束缚和深层次矛盾亟待破题。困难需要一个一个去克服，问题需要一个一个去解决。只要我们解放思想、大胆探索，忍受阵痛、破除阻力，就一定能够释放改革红利、创新活力。

我们务必放大优势，乘势快进。国家承接产业转移示范区、长江中游城市群、洞庭湖生态经济区三大战略机遇汇集，正在转化为振兴荆州的强大磁场；国家依托长江建设经济新支撑带，扩大沿江内陆开放开发，将为振兴荆州注入强大动力。只要我们向前展望、超前思维、提前谋局，就一定能够把战略优势转化为发展优势。

我们务必顺应趋势，好中奋进。国内经济主动转型调整，进入增长速度换挡期，升级增效已经成为“主旋律”。荆州作为湖北之腰，支撑支点建设，既要做大总量，更要提升质量。只要我们坚定不移实施壮腰工程，稳住心神、保持定力、不急不躁，在稳进中转型、在扩量中提质，就一定能够实现目标进取、发展进位、全面进步。

今年，我们将认真抓好以下五个方面的工作：

（一）以改革促转型，提升产业发展质量。抢抓全面深化改革带来的发展“窗口期”，壮大实体、筑牢根基，主动转型、有效整合，从单打独斗的“企业效益”向集群发力的“产业效应”转变。

加快新型工业化步伐。把强工业作为壮腰工程的主攻方向，坚持扩量与提质并重，改造传统产业与壮大新兴产业并重，提升工业整体素质，力争第二产业比重提高2个百分点以上。加快支柱产业改造升级。以完善产业推进机制为重点，改革财政扶持方式，充分发挥重大产业发展基金、政府引导基金、天使基金和企业生产调度资金的撬动作用，力争装备制造业突破500亿元。以科技创新为动力，依托伟特电子、同洲电子、华讯方舟、711厂、明德科技等企业和项目，扩大“中国天谷”聚集效应，打造中部地区电子信息产业基地。以择优培优、引领示范为立足点，积极培育高端装备制造、新材料、新能源和生物制药等战略性新兴产业。加快工业企业扩量升级。强化市场在配置资源中的决定性作用，落实结构性减税政策，开展科技型“小巨人”、“助保贷”和融资担保业务，推进中小企业成长工程，让更多“小树苗”长成“参天大树”，力争新增规模以上工业企业150家。积极发展交叉持股、相互融合的混合所有制企业，鼓励企业靠大引强、兼并重组，二次改制、二次创业，力争中兴能源、福娃集团等企业销售收入过100亿元。引导企业更加自觉运用信息技术提升企业管理水平，更加注重创新、创牌、创造，扩大“荆州智造”的影响力和竞争力。加快县域工业提档升级。进一步优化和落实支持县域经济发展的政策措施，引导县市区依托优势资源，规划产业布局，做强特色产业。重点支持荆州区石油机械和造纸包装、沙市区机电汽配和针纺服装、江陵县煤电能源、松滋市白酒酿造、公安县汽车零部件、石首市医药化工、监利县粮食加工、洪湖市水产加工等产业发展，推动县域经济争先进位。

提高农业现代化水平。坚持用工业化理念强农业，用市场化手段富农民，用创新的办法美农村。以特色化提升农业优势。集中连片开展土地整理和高标准农田建设，推进标准化示范县建设，以更大的担当和力度创建“百亿斤粮食大市”，力争今年粮食总产突破80亿斤。发展工厂化种植和规模化养殖，做大优质水稻、特色蔬菜、名优水产等产业板块。推广绿色农业、循环农业模式，逐步改变“靠化肥堆、靠农药保”的生产方式。建立农产品质量可追溯制度，开展农产品电子认证、种养殖业在线监测，确保“舌尖”上的安全。以产业化带动农业发展。稳步推进土地适度规模化经营，引导工商资本到农村发展适合企业化经营的现代种植业，向农业输入现代生产要素和经营模式，解决“谁来种地、怎么种地、种了怎么办”的问题。以规范发展、创新模式、做实做强为重点，把更多的财政项目资金直接投向龙头企业、合作社、合作联社，鼓励新型农业经营主体跨区域、跨行业发展。开展农社对接、农超对接、农金对接，拉长产业链、延伸价值链，加快构建复合型现代农业经营体系。继续实施“四个一批”工程，培育一批产值百亿元以上的农产品加工园区、加工企业，引导企业走全产业链发展之路，推进农产品由初加工向精深加工转变，力争全市农产品加工业产值与农业总产值之比达到2.2∶1。以改革创新拓展农业空间。抓好江陵县农村土地承包经营权确权登记颁证试点。规范有序推动所有权、承包权、经营权三权分离，让土地像资本一样流动起来。稳妥推进农民住房财产权抵押、担保、转让，赋予农民更多财产权利。健全农产品网络交易平台，发展涉农“电商”，用信息化技术缩短农民与市场的距离。进一步理顺国有农场管理体制，推进分离办社会职能改革。在改革过程中，尊重群众首创精神，发掘基层鲜活典型，以点带面，寻求突破。

促进服务业转型发展。牢牢把握扩大内需这一战略基点，加快推进产品创新、服务创新、模式创新，将内需潜力转化为助推发展的重要动力。全面提升文化旅游产业水平。更加珍惜城市文脉和历史遗产，处理好保护、传承与开发的关系，守护好我们共同的精神家园，推动荆州文化品牌到文化产业的转型跨越。加快中心城区文化旅游资源整合和总体开发利用，启动包括关公文化园在内的荆州古城5A级景区创建，力争用3年左右时间，将海子湖生态文化旅游区打造成中国楚文化展示区、国家大遗址保护示范区、国家文化产业示范区。完善提升楚王车马阵、洪湖岸边是家乡、松滋洈水、石首天鹅洲等景区景点，积极参与江汉运河生态文化旅游城镇带建设，唱响楚文化、三国文化和水文化品牌。积极对接高铁和长江、汉江沿线城市，发展旅游联盟，开拓客源市场，力争全市旅游综合收入增长20%。全面提升商贸物流产业水平。积极发展临港、临铁产业，加快建设一批现代物流园区和物流配送中心，突破性发展供应链直供直销、物流总部等新兴业态。推动信息化与其他行业深度融合，发展移动互联网、云计算、物联网等新一代信息技术产业，加快建设智慧城市。实施第二批现代服务业综合改革试点，落实鼓励消费的信贷和财税政策，扩大社区养老、体育健身、文化创意、信息等服务消费。适应电子商务迅猛发展的趋势，倒逼传统商业转型升级。全面提升金融服务水平。强化政银企沟通协调机制，加强征信体系建设，解决金融信息不畅、企业信用不够问题。推进本地企业上市和上市企业再融资，支持在“新三板”和区域股权交易市场托管、挂牌、融资，提高直接融资比重。深化农信社改制重组，规范发展村镇银行、社区银行、融资性担保、专业保险等新型金融机构，让金融服务创新更好地助推经济社会发展。

（二）以改革优环境，提升发展内生动力。健全创新驱动发展的体制机制，培育市场公平竞争的土壤，增强生态环境承载能力，努力打造荆州“软实力”，为加快振兴提供“硬支撑”。

充分激活创新要素。以国家、省级工程技术中心和创业中心为平台，以构筑创新链、完善资金链为重点，建立健全区域创新体系，消除科技创新的“孤岛现象”。突出企业创新的主体地位，使企业真正成为投入、研发和应用主体。积极申报国家和省科技计划项目，继续开展企业专利“扫零”工作和“重点企业知识产权产出培育工程”。支持各开发区与高等院校、科研院所建立产业技术创新战略联盟，建设高科技产业园和高校产业园，努力把科教资源优势转化为现实生产力。建立科技型中小企业风险投资机制，促进科技成果资本化、产业化。继续实施“金凤人才”计划，加强院士专家工作站建设与管理的政策支持，更加注重企业家培养。健全技术要素参与分配激励机制，引导人才向经济一线流动。

着力优化市场环境。全面清理不利于市场公平的“土政策”，探索“负面清单”管理模式，以“非禁即准、宽进严管”为原则，消除对非公有制经济发展的各种附加条件和隐性壁垒，实施市场主体增量行动，让“经济细胞”加快繁殖、充满活力。以管资本为主加强国有资产监管，支持非公有制资本依法参与国有企业和经营性事业单位改制重组。继续深化国有企业公司制、股份制改革，用市场化手段推动企业建立现代管理制度。加快引进有资质、有实力的中介机构，推进中介机构脱钩改制，坚决打破唯一性、指定性。进一步规范房地产、建筑市场。以一流的政务服务，为市场主体发展营造良好外部环境。

积极创建生态城市。牢固树立尊重自然、顺应自然、保护自然的理念，鼓励发展绿色、低碳、循环经济，创建国家生态城市，建设美好家园。以改革管理体制为抓手，建立健全投资项目环评、能评制度，严控污染物排放总量，严格淘汰落后产能，严惩破坏生态环境行为。以创新植树造林机制为抓手，实施“绿满荆州”行动，打造沿江、沿湖、沿河生态绿化带。以“四城同创”为抓手，加大城市污水、垃圾处理等基础设施建设，推进护城河、西干渠截污、四湖总干渠治污和城区水系连通，实施雨污分流工程，彰显水乡园林城市的生机与灵气。加快洪湖、长湖生态修复，推进国家湖泊生态环境保护试点项目建设，鼓励创建国家级、省级湿地公园。加强城市规划控制，科学预留间距和风道、绿道，推进生态治水、管城、护绿，改善城市环境。高度重视大气污染防治问题，严格控制建筑工地、城市道路扬尘污染，全面实施机动车尾气排放管理，限制城区燃放烟花爆竹，推进城区燃煤锅炉改造，开展秸秆禁烧和综合利用试点。实施清洁家园行动，治理农村面源污染和规模化养殖污染，多栽树、不填湖、少拆房，让人们望得见蓝天、看得见碧水，忘不了故土、记得住乡愁。

（三）以改革扩投资，提升开放开发水平。以更大的决心和韧性，坚定不移地敢开放、真开放、全开放，夯实投资底盘，引进投资主体，建好投资载体。

持之以恒抓招商。进一步梳理、规范招商政策，明确产业定位，绘制“产业图谱”，承接产业转移，加快引进一批产业链、龙头型的大项目、好项目，力争实际到位资金突破1000亿元。整合招商资源，强化驻点招商、定向招商、园区招商、以商招商等方式。用心呵护“草根经济”，做到内商外商公平待遇、内资外资同等重视，促进外来资本与本地产业有效对接。改革招商激励和考核方式，建立招商项目评估机制，注重税收贡献度、产业关联度和环境承载度考量，决不为眼前利益给历史留下败笔。

大干快上建项目。健全完善领导包挂、跟踪督办工作机制，着力解决融资难、征迁难、招工难问题，力争固定资产投资突破1700亿元。工业方面：加快推进华讯方舟、骏马涂布烟卡纸、中哈能源等26个投资10亿元以上的重大项目建设和100个投资千万元以上的技术改造项目。交通方面：确保江南高速公路建成通车，完成荆松一级公路路基工程，力争东岳庙至卷桥高速公路年底建成；加快推进江汉平原货运铁路立项和“北煤南运”铁路、洪监高速、武汉城市圈环线洪湖段、潜石高速公路江陵段、江北高速等项目建设，开工建设荆州区旅游公路、沙市至公安高速公路。水利方面：全面完成引江济汉通水、通航工程，加快推进荆江大堤综合整治、荆南四河堤防加固、四湖流域综合治理、病险水库除险加固和大型泵站改造等项目建设；突出民生水利，改革小水利投融资体制，加快解决水利建设“最后一公里”问题。

加快拓展工业园区。按照“先规划后建设、先地下后地上、先配套后招商”的理念，全面提升园区综合承载功能。举全市之力支持荆州开发区转型升级，丰富“国牌”的内涵和含金量。统筹推进国家级承接产业转移示范区建设，加快推进现有园区扩容提质，推进布局集中、产业集聚、用地集约，力争荆州开发区新建园区10平方公里、各县市区新建园区5平方公里以上。支持华中农高区创建国家级农业高新技术产业开发区，力争用3年左右时间完成农高区核心区建设。创新园区建设经营机制，引入公司化管理方式，推动开发区改版。探索园区产业供地和企业进退机制，为更多优质项目入驻腾出发展空间。

（四）以改革兴城乡，提升城乡整体水平。坚持以人为本、产城融合、城乡互动，积极稳步推进新型城镇化，加快融入长江中游城市群，建设美丽荆州、幸福家园。

推进新区开发和旧城改造。像经营企业一样经营城市，做大城投融资平台，垄断土地一级开发市场，推进土地开发融资权拍卖试点，用市场化方式筹措资金。加快以荆州开发区为主的工业新城、以华中农高区为主的农高新城、以海子湖新区为主的文化旅游新城建设，带动古城人口疏散和旧城改造。抓紧实施沙市中学、荆州中学和荆州中心医院搬迁工程。积极有序推进中心城区合理布局和规模扩张，不重复投资、不无效投资、不盲目负债。提升城市建设管理科学化水平，坚持一张蓝图绘到底、管到底、抓到底。

加快建设荆江城镇带。以战略眼光编制城乡全域规划和专项规划，推进经济社会发展计划、国土利用规划、产业发展规划与城乡建设规划“四规合一”。因地制宜、量力而行，鼓励创建国家级或省级园林城、环保模范城、卫生城、生态城、文明城等，提升城市功能和文化品位。找准“四化同步”发展的结合点、创新点，探索市场驱动、政策推动、农民主动的城镇化发展模式，推进经济发达镇行政体制改革和11个“四化同步”试点乡镇（场）建设，力争每个县市建成1-2个产业集聚、人口集中的中心镇或特色镇。逐步放开建制镇和县市城区落户限制，优先解决已到城镇就业的农业转移人口落户问题。规范农村建房审批管理，统筹城乡一体化发展，把公共服务与城镇化、新农村建设有效对接，保护好、建设好有历史记忆、地域特色、民俗文化的美丽乡村，推进就地、就近城镇化。

全力以赴办好省运会。省运会首次在荆州举办，这是全市人民的荣耀，也是一份沉甸甸的责任。探索政府支持、市场运作、社会赞助、群众参与的办赛机制，集全民之智、借全省之力，办一届特色鲜明、节俭安全、精彩圆满的运动会，以此凝聚新力量，展示新形象，助推新发展。加快体育中心建设、奥体中心改造、配套场馆升级，全面完成场馆建设任务。深入开展城市环境整治和文明创建，积极开展全民健身活动，以主人翁姿态和“洁、绿、亮、美、畅”的城市形象迎接八方嘉宾。深化城管体制改革，推进执法力量下沉，综合施治交通拥堵、沿街为市等顽疾乱象，确保城市管理常态化、长效化。

（五）以改革惠民生，提升社会保障能力。牢固树立“民生决定目的”理念，着力“保基本、补短板、兜底线”，帮困解难见真情，春风化雨惠民生。

扩大公共财政支出。深化财税体制改革，实施全面规范、公开透明的预算制度，建立健全预算绩效管理和均衡预算支出机制，提高财政收入质量，把更多新增财力投向创业就业、社会保障、社会救助、扶贫开发等民生事业，让人民群众感受到党和政府的温暖。统筹抓好服务企业用工和城乡劳动者就业工作，深化创业型城市创建并逐步向县市延伸，促进大中专毕业生、农业转移人口、失地农民等群体多渠道就业，支持青年、妇女和下岗失业人员自主创业，引导外出务工人员返乡就业创业。积极对接国家社会保险改革方案，进一步探索机关和企事业单位养老保险改革新途径，把社会保障服务平台延伸到村，实现社会保障全覆盖。推进社会救助主体多元化，引导社会资本办社会福利和社区养老机构。提高城乡低保标准和“五保户”供养水平，重视解决农村“三留守”问题。积极发展慈善、老龄、红十字和残疾人事业。加强农村“三资”管理，稳妥化解村级债务。开展造血式项目扶贫开发，整村推进重点老区、民族乡和插花贫困乡镇扶贫。深入推进价格改革，完善价格调节基金制度和物价补贴联动机制。实现城乡居民收入和经济发展同步，让老百姓腰包“稳增长”。

提高公共服务质量。把教育放在优先发展的位置，用改革提质量、促公平、保均衡。更加重视农村教育，健全家庭经济困难学生资助体系，优化中小学校布局，探索建立校长、教师跨区域交流轮岗机制，用信息化手段扩大优质教育覆盖面；更加重视素质教育，从学前教育抓起，巩固“两基”成果，切实减轻学生课业负担，让爱的教育伴随每一个孩子健康、快乐成长；更加重视职业教育发展，促进职业教育在专业设置、办学规模、内涵建设上与社会需求相适应，支持荆州职业技术学院建成全省一流示范性高等职业院校。支持长江大学及其独立学院等在荆高校发展，深化校地合作机制，开创高校建设新局面。加大公园、公交、公厕建设投入，优化配置停车位，让市民出行更加便捷。统筹推进医疗服务、公共卫生、药品供应等综合改革，健全网络化城乡医疗卫生服务机制，合理布局和配置医疗资源。继续开展综合治理，提高疫区血吸虫防治水平。推进国有经营性文化单位转企改制，建立文化惠民工程与群众文化需求有效对接机制，加快政府由办文化向管文化转变。落实“单独二孩”生育政策，综合治理出生人口性别比，促进人口长期均衡发展。继续做好第三次经济普查。加强档案馆舍与信息化建设。认真抓好国防动员、民兵预备役、人民防空工作，支持驻荆部队建设，进一步做好外事侨务、台湾事务、民族宗教、防震减灾、移民、盐务、邮政、供销、气象、史志、保密、无线电等工作，努力维护妇女和儿童权益。

增强社会治理能力。推进管理集成、力量下沉，健全覆盖城乡的网格化管理体系。继续开展精神文明创建，认真抓好“六五”普法和科普工作，提升市民文明素养。处理好维权与维稳的关系，规范执法行为，强化司法援助，打造“法治荆州”。发挥社会团体参与社会治理的作用，开展社区矫正工作，强化流动人口、特殊人群服务管理。探索建立政府、社会、企业多方参与的公共安全监管模式，调整食品、药品安全监管体制，突出抓好交通、消防、校车和危化品等重点领域安全隐患整改，健全应急管理体系，确保不发生重特大事故。完善基层信访工作网络，推行网上受理信访制度，建立畅通有序的诉求表达、矛盾调处、权益保障机制。深入推进“平安荆州”建设，打造立体化社会治安防控体系，依法防范和惩治违法犯罪活动，让公序良俗得以确定，让公平正义得到保障。

努力为民办实事。新增城镇就业7.5万人，扶持创业5000人，带动就业2万人，发放小额担保贷款2.5亿元；免费培训新型农民2万人，转移培训农村贫困劳动力1600人；新增住房租赁补贴1170户，建成保障性住房1.74万套，在保证建筑质量的基础上，提高舒适度；解决农村30万人的饮水安全问题；推进“天网”工程建设，架设A类视频监控探头7400个；新建农村互助照料活动中心220个；为2.8万对农村夫妇提供免费孕前优生健康检查，对5万名农村留守妇女进行“两癌”免费检查，向有创业意愿的农村留守妇女提供小额担保贷款4000万元；投资1200万元，帮助55家中心城区企事业单位改用清洁能源、淘汰燃煤锅炉；为困难群众和农民工办理法律援助案件2200件，办理法律援助事项2.2万件以上；免费为100名贫困下肢残疾人安装假肢，为200名贫困白内障患者实施复明手术，为1000名贫困精神残疾人提供基本控制药物，为200名0-6岁贫困残疾儿童实施抢救性康复，为中心城区100名残疾人进行家居无障碍设施改造，为2000名0-14岁残疾儿童和残疾人家庭子女实施集善残疾儿童助养项目；中心城区购置公交车130台，新建2处公交枢纽场站；建成9个放心粮油配送中心和100家放心粮油连锁店。民生无小事，我们将用力、用心、用情去办，努力让老百姓看得见、摸得着、得实惠！

三、加强政府自身建设

深化改革没有退路可言，加快振兴没有捷径可走。我们必须以更加开明的思想、更加开放的胸怀、更加开拓的精神，打好一场政府自身改革的硬仗，以变应变、提能善政、善做善成。

（一）转变理念谋发展。树立改革发展的理念，充分利用全面深化改革的倒逼机制，勇于突破利益固化的藩篱，坚决破除体制机制弊端，向改革要红利，用改革增优势，以改革聚能量。树立创新发展的理念，敢于破除传统思维定势和工作方式，以思想的大解放换取荆州的大发展。树立科学发展的理念，致力打基础、管长远，致力优环境、利民生，多做“前人栽树、后人乘凉”的事情，多做“润物细无声”的事情，把工作的着眼点、着力点调准校好，坚决纠正单纯以经济增长速度评定政绩的倾向，决不因今天的短视影响明天的发展。

（二）转变职能增活力。严格执行中央“只减不增”的规定，推进“大部制”、“大科室制”和事业单位分类改革，打破编制“增减循环”，形成大服务、大管理格局。制定“权力清单”和“责任清单”，厘清政府与市场的边界，该管的坚决管好，不该管的坚决放活，用政府权力的“减法”换取市场活力的“加法”。尊重市场规律，用好市场机制，进一步清理、调整和减少行政审批事项，解决政府越位、错位、缺位问题。切实把政府职能转变到公共服务、市场监管、社会管理和环境保护上来，不能既当“运动员”、又当“裁判员”，而要当好“引航员”、“服务员”，充分发挥“有效市场”和“有为政府”的作用。

（三）转变方法强治理。善用法治思维和法治方式，推进政府治理体系和治理能力现代化。更加注重依法行政，自觉接受人大法律监督、政协民主监督和社会舆论监督，强化政府层级监督和监察、审计等专门监督；完善行政执法体制，建立政府法律顾问制度，健全重大事项决策机制，把公众参与、专家论证、风险评估、集体讨论和合法性审查作为必要程序，防止因决策失误贻误发展。更加注重政务公开，规范政府工作流程，加强电子政务建设，推进决策公开、服务公开、结果公开。更加注重绩效管理，强化大督办、大协调机制，引进项目管理方式，每一项具体工作都制定“路线图”、“时间表”，有效传导压力，打造落实链条，确保政令畅通、令行禁止。

（四）转变作风干实事。根往下扎得越深，树才能长得越茂。结合新一轮“三万”活动，下基层、接地气，密切血肉联系，走好群众路线。把更多的时间和精力放在招商引资、项目建设、征迁安置、信访维稳等一线工作上来，踏石有印、抓铁留痕。强化治庸问责，治理“中层梗阻”，破除“遇事找熟人、办事讲关系”现象，着力解决不作为、慢作为、乱作为等问题，让基层企业和群众少跑路、少受累。持之以恒反“四风”，坚决与陈规陋习作斗争，从节俭办事和探索公车改革等具体事情抓起，坚持公款公用、专用、节俭用、透明用，压缩一般性支出，用政府的紧日子换取群众的好日子。认真落实党风廉政建设“一岗双责”制度，推进源头治理、制度反腐，敢于“亮丑”、敢于“亮剑”，对违法违纪行为“零容忍”，以实实在在的反腐成效向全市人民交上一份合格的答卷。

各位代表！有梦就有动力，有梦就要坚持，有梦就能出彩。梦在前方，路在脚下。让我们更加紧密地团结在以习近平同志为总书记的党中央周围，在市委的坚强领导下，高举改革大旗，凝聚振兴力量，迈开坚实步伐，一步一个脚印，一年一个台阶，为打造湖北经济增长“第四极”、实现荆州振兴梦想而不懈奋斗！
